# The Beauty of Bacteriophage T4 Research: Lindsay W. Black and the T4 Head Assembly

**DOI:** 10.3390/v14040700

**Published:** 2022-03-28

**Authors:** Andreas Kuhn, Julie A. Thomas

**Affiliations:** 1 Microbiology, Institute of Biology, University of Hohenheim, 70599 Stuttgart, Germany; andreas.kuhn@uni-hohenheim.de; 2 Rochester Institute of Technology, Thomas H. Gosnell School of Life Sciences, College of Science, Rochester, NY 14620, USA

**Keywords:** T4 bacteriophage, head assembly, DNA packaging, internal proteins, giant phage

## Abstract

Viruses are biochemically complex structures and mainly consist of folded proteins that contain nucleic acids. Bacteriophage T4 is one of most prominent examples, having a tail structure that contracts during the infection process. Intracellular phage multiplication leads to separate self-directed assembly reactions of proheads, tails and tail fibers. The proheads are packaged with concatemeric DNA produced by tandem replication reactions of the parental DNA molecule. Once DNA packaging is completed, the head is joined with the tail and six long fibers are attached. The mature particles are then released from the cell via lysis, another tightly regulated process. These processes have been studied in molecular detail leading to a fascinating view of the protein-folding dynamics that direct the structural interplay of assembled complexes. Lindsay W. Black dedicated his career to identifying and defining the molecular events required to form the T4 virion. He leaves us with rich insights into the astonishingly precise molecular clockwork that co-ordinates all of the players in T4 assembly, both viral and cellular. Here, we summarize Lindsay’s key research contributions that are certain to stimulate our future science for many years to come.

## 1. Introduction

During the last 50 years of research on the morphogenesis and packaging of the T4 phage head, an enormous amount of information regarding this fascinating process has been obtained. Lindsay W. Black was a major contributor to many of these discoveries, spending much of his career unravelling intricate, molecular details of T4 head assembly. Lindsay was acutely aware that, despite the existing knowledge, there was still much to be learned regarding the exact functions and interactions of many of its molecular participants. He recognized the interdependency of many of the T4 head assembly steps, and that phage assembly is a domino-like process guided by sequential protein-folding events. The complete understanding of each step requires data acquired via multiple approaches. Consequently, Lindsay’s research is notable for its incorporation of genetics, electron and fluorescent microscopy, proteomics, various molecular and many biochemical analyses. Lindsay also participated in many fruitful collaborations, and we refer the reader to contributions in this issue by several of Lindsay’s long-time collaborators [1]. In this article, we highlight Lindsay’s contributions to our understanding of DNA packaging and the major steps in T4 head assembly, and focus on the process of head maturation. We will also highlight how he exploited T4 head assembly mechanisms biotechnologically, e.g., to incorporate novel proteins into the T4 head. We will conclude this review by emphasizing the importance of T4 as a model for understanding head assembly and maturation in related phages.

## 2. From a Dodecameric Ring to a Complex DNA-Full Prolate Icosahedron—The Molecular Assembly Line That Produces the T4 Head 

### 2.1. Overview of Major Steps in T4 Head Assembly

**Figure 1:**
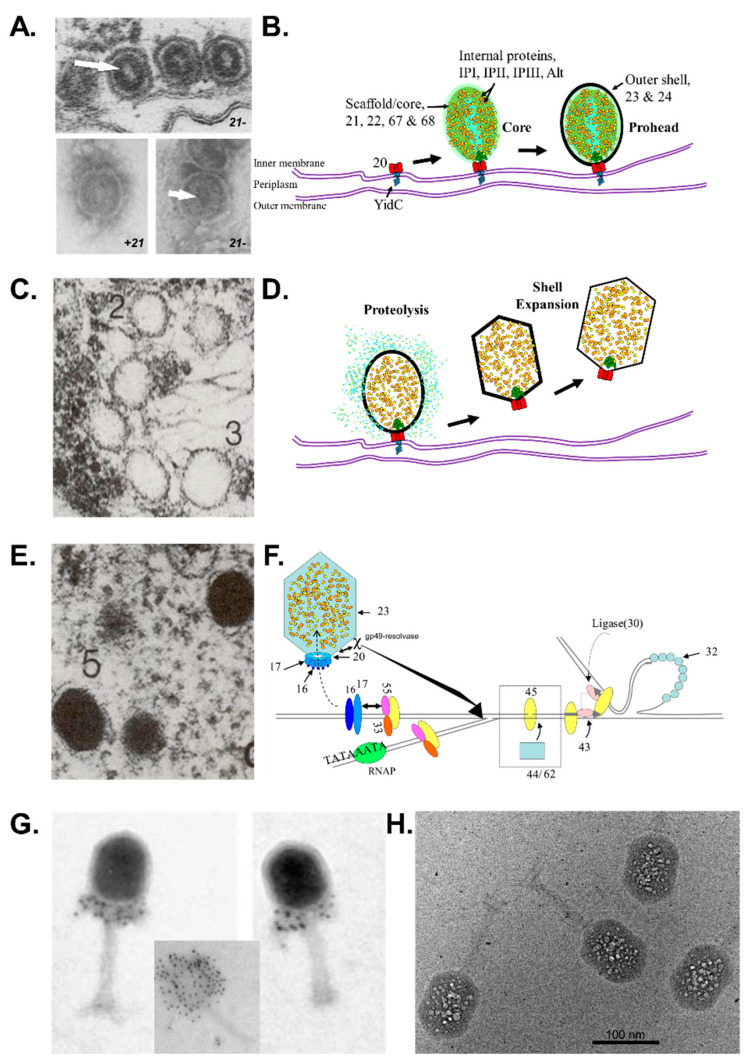
Overview of the major steps in T4 phage head assembly. (**A**,**B**) Assembly initiates via the formation of a protein core anchored to the *E. coli* inner membrane via the portal, and around which the major capsid protein concomitantly assembles. Only in the absence of gp23 can naked cores be observed. (**A**) Electron microscopy of proheads produced by 21- mutants in vivo (upper, thin section) and in vitro (lower). White arrow indicates a central hole in proheads assembled in the absence of the prohead protease (adapted from van Driel, Traub and Showe [6]). (**C**,**D**) Proteolytic maturation involves cleavage and removal of scaffold/core proteins as well as the propeptides of the internal and shell proteins, release of the prohead from the inner membrane and semi-expansion of the shell. (**E**,**F**) Packaging of the genome into the prohead occurs via the action of the main packaging proteins, TerS (gp16) and TerL (gp17). (**C**,**E**). Electron microscopy of thin sections of wild-type T4-infected *E. coli* (reproduced from Black and Thomas [7]). (**F**) Scheme of how DNA packaging in vivo is integrated with late transcription, and DNA replication (reproduced from Black and Peng [8]). (**G**) Recombinant Δhoc phage particles after immuno-gold labelling with an anti-Hoc antibody (inset WT phage particle). The visualization of the “gold necklace” provided evidence that the portal structure contained fusion proteins (gp20-Hoc). Confirming the recombinant phenotype was important, as these particles had a central role in refuting the rotary portal packaging model (reproduced from Baumann, Mullaney and Black [9]). (**H**) Cryo-electron micrograph of a T4 Alt mutant imaged after the eighth exposure of a dose series of 16.5 el/Å^2^ per exposure). The bubbles are generated from the internal proteins, which are inferred to be randomly positioned within the DNA, but excluded from a zone of about 100–110 Å directly under the outer shell (reproduced from Wu et al. [10]).

T4 head assembly initiates at the cytoplasmic surface of the inner membrane of *Escherichia coli* by the interaction of the gp20 portal protein with the membrane insertase YidC [2] (Figure 1). The resulting dodecameric ring structure forms the foundation onto which a protein-only structure, the “core”, forms and acts as a scaffold to direct the correct assembly of the prolate exterior shell [3,4]. Concomitantly with core formation, or immediately after, hexagons of the major shell protein gp23 and pentagons of gp24 assemble around the core, leading to the formation of a “prohead” (called “prehead” in early work). The core contains the proteins gp21, gp22, gp67 and gp68, together with the internal proteins Alt, IP1, IPII and IPIII. Proheads undergo proteolytic maturation by gp21, detach from the membrane, and the capsid shell expands to its final symmetry [2]. DNA is then packaged into the capsid via interactions of the terminase with the portal protein [5]. Once packaging is completed, a neck structure composed of gp13 and gp14 assembles onto the portal, allowing the addition of the tail and tail fibers. These steps in T4 assembly are typically referred to individually (e.g., assembly, maturation, packaging), but it is important to recognize that each is interlinked. Hence, each step is somewhat dependent on the former and/or concomitant steps, but also part of the overall process.

### 2.2. Lifting the Secrets of T4 DNA Packaging

For most of his scientific career, the major focus of Lindsay Black’s research was on the DNA packaging process of the bacteriophage T4. DNA packaging is initiated on concatemeric DNA by an enzyme called terminase, consisting of gp16 (TerS) and gp17 (TerL). Whereas the endonucleolytic function of gp17 became evident early, the role of gp16 remained unclear for some time because it is not essential in vitro [11]. TerL terminase binds the DNA without a sequence specificity, in contrast to the T4-like phage IME08 that recognizes a consensus site and produces a staggered cut of the replicated phage DNA [12]. After the T4 terminase has bound to the concatemeric DNA, it is transferred to the portal of the proheads. The proheads are present in the cytoplasm of the cell as they are released from the host membrane shortly after head maturation (see below).

The prominent role of gp20 in packaging was detected by Hsiao and Black early with the cold-sensitive mutant *cs*20 [13]. Infections at the non-permissive temperature led to the accumulation of empty capsids in cells, which were apparently packaged when the infected culture was shifted to the permissive temperature. From these experiments, it was concluded that gp20 has two distinct functions: one is to initiate the assembly of proheads at the membrane, and the other in the DNA packaging process. This role for the portal was corroborated by Quinten and Kuhn via a T4 mutant whose gp20 protein has an amino-terminal truncation of 14 amino acid residues [2]. The short gp20s protein produced by this mutant facilitated the assembly of proheads; however, these proheads were hindered in the DNA packaging process, showing an accumulation of packaging intermediates.

**Figure 2:**
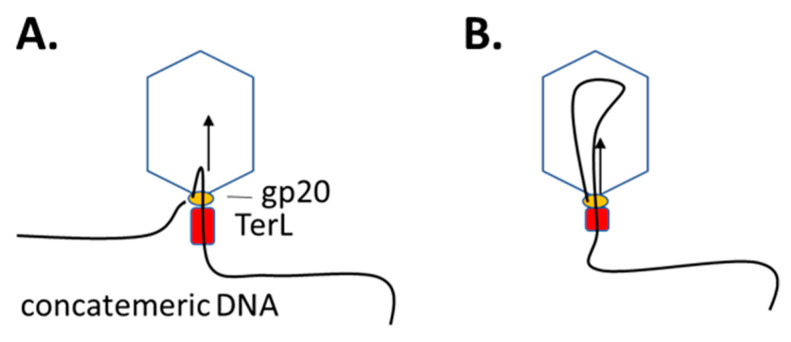
Packaging of concatemeric DNA into the T4 head. One end of the DNA is held at the gp20 portal, whereas the free strand is funneled through the portal as a loop structure (**A**) by sequential movements of the TerL subunits and hydrolysis of ATP (**B**).

The concatemeric DNA molecule that is bound at the portal by the terminase is sequentially filled into the capsid, presumably as an elongated loop structure, since the two ends of the packaged DNA stay at the portal outside of the head [14]. To unravel the mechanistic details of this fascinating process, one needs to closely follow the molecular dynamics of all of the players, i.e., the terminase, the DNA and the portal (Figure 2). A major step forward in understanding how the terminase moves the DNA into the capsid was accomplished by the crystal structure of TerL [15]. The overall domain structure of TerL is broadly conserved in many tailed phages, and comprises three domains: an *N*-terminal ATPase domain similar to P-loop ATPases/helicases, that is connected to a *C*-terminal endonuclease domain by a central hinge region [16]. The crystal structure of TerL showed that the substrate DNA is bound to the translocation domain as well as to the ATPase subdomain II. Interestingly, TerL exists in two conformational states—a tense and a relaxed state—that is controlled by ATP.

The development of an in vitro packaging system in T4 made it feasible to functionally follow the molecular events using biophysical methods. Purified proheads are first mixed with purified gp17, then the substrate DNA (T4 genomic, plasmid or other) is added and subsequently efficiently packaged into the proheads in the presence of ATP [8,17]. In Lindsay’s lab, fluorescence correlation spectroscopy was applied with this system to follow the binding of the substrate DNA where the 5′ end of the DNA was labelled with rhodamine [18]. Using the same assay, Förster resonance energy transfer (FRET) was observed when GFP, incorporated into the capsid (see below), was used as an exciton donor and combined with DNA fragments that were labelled with Texas red and served as an acceptor dye. This allowed the follow-up of the DNA packaging process in real time.

The hypothesis that the rotation of the portal was involved in the packaging process had been favored for several decades, in part because it would explain why there is a symmetry mismatch (12:5) at the portal vertex, and seemingly supported by the turbine-like structure of the portal itself [19,20]. The rotary portal hypothesis was difficult to test, and led to the elegant experiment designed by Lindsay, Julienne Mullaney and Rick Baumann to test it. They exploited their ability to create active recombinant phages with fusion proteins assembled into the virion, and engineered particles with portal proteins that had either a *C*-terminal fusion (gp20–GFP, gp20–Hoc) or an *N*-terminal fusion (GFP–gp20 and Hoc–gp20) [9], with Hoc being the “Highly immunogenic outer capsid protein”, a dispensable protein that binds to the capsid hexons after expansion of the shell [21]. The logic behind this experimental design was that a Hoc–gp20 fusion protein would prevent the rotation of the portal relative to the outer shell, i.e., if rotation was necessary for DNA packaging, the resulting phage particles would not contain DNA and be non-viable. Alternatively, if rotation was not required for DNA packaging, then the particles should contain DNA (and be viable). Particles with the Hoc–gp20 fusion proteins were used in an in vitro packaging assay, which showed that, in fact, DNA was able to move into the capsid. The localization of the Hoc–gp20 fusion proteins in the portal region was confirmed using immuno-gold labelling with an anti-Hoc antibody (Figure 1G). Shortly after, Hugel et al. [22] acquired evidence supporting that the portal did not rotate during packaging in φ29 via following the orientation of single fluorophores attached to the portal motor connector during DNA packaging using single-molecule force spectroscopy with polarization-sensitive single-molecule fluorescence.

Cryo-electron microscopical studies [15] showed that the gp17 terminase is a pentameric structure that surrounds the DNA and presumably moves the helix by the sequential interactions of its protomers. This “pushing” then leads to a compression of the DNA towards the portal, resulting in DNA movement and packaging into the head. The compression might also lead to a structural change of the DNA from the B-form to the A-form, which is less hydrated [23,24]. This observation was corroborated by FRET experiments. The distance of the terminase relative to the portal during packaging was followed by single molecule smFRET [5]. The alanyl residue A316 in the clip region of gp20 was labeled with Alexa488, whereas gp17 was labeled with fluorescein at the *C*-terminus. The FRET measurements of these gp17-bound proheads revealed a distance of the two dyes of 7.5 nm during packaging. Based on these experiments, a time-resolved analysis of intramolecular movements of the fluorescently labeled DNA versus the terminase has been recently investigated using total internal reflection fluorescence (TIRF) [25].

### 2.3. Mysterious Small T4 Internal Head Proteins

In contrast to many phages, the mature head of the T4D phage contains not only the packaged 169 kb genome (~171 kb with terminal redundancy [26]), but also many internal proteins and the processed fragments of the core proteins besides the protective outer shell gp23* and gp24* (Table 1). The head proteins (gp21, gp22, Alt, gp67, gp68 and three internal or IP proteins IPI, IPII and IPIII) are incorporated into the prohead core prior to the addition of the outer shell and DNA packaging. During infection, many of the IP proteins and Alt are ejected into the host cytoplasm, suggesting that at least some of them have roles in host take-over. Such a role was determined for Alt, an ADP-ribosyltransferase that modifies the host RNA polymerase to enhance its specificity for T4 promoters, however, despite this role, Alt is not essential [27,28,29]. 

The internal proteins IPI, IPII and IPIII intrigued Lindsay throughout his career, and one of his earliest studies determined their molecular masses (~8, 10 and 20 kDa, respectively) by purifying them from a phage suspension in which the particles had been ruptured by osmotic shock [31]. Quantitative calculations suggested that about 300 copies of each of these basic proteins are present per phage head, and this provoked the assumption that they interact with the phage DNA. Indeed, under low-salt conditions, the IP proteins did bind to DNA [31], but their role in the phage head remained elusive.

To interrogate the function of the IP proteins, three mutants were isolated after hydroxylamine treatment of the T4 DNA, each having an amber mutation in one of the three IP genes and a mutant IP^0^ with all three amber mutants combined [32]. The isolation of these mutants revealed the IP proteins were dispensable for T4 growth, i.e., their genes were not essential in the traditional sense, as the mutants could grow when tested on the standard non-permissive *E. coli* strains used for T4 genetics. This finding was unexpected, as the large amount of these proteins in the capsid had led to the inference that they must be important. Intriguingly, further study of the IPIII mutant revealed that its rate of head assembly was reduced by about half, as judged by the rate of proteolysis of head proteins (see below), and the burst size was about 40% of normal [32]. These characteristics of the IPIII mutant were shown not to be dependent on the presence or absence of either IPI or IPII in the head after analyses of recombinant phages of the various IP mutations, including a mutant in which the three IP genes each had an amber mutation (IP^0^) [33]. Electron microscopy confirmed that there were aberrations in head assembly in both the IPIII^−^ and IP^0^ mutants, as revealed by increased accumulations of polyheads [33]. These findings suggested a role for IPIII in T4 head morphogenesis, possibly together with the core protein, gp22 [34]. The co-purification of the internal proteins with gp22 corroborated their interaction with gp22 [33]; however, the exact role(s) of IPIII (in head assembly and/or host take-over) remains to be solved, as does the role of IPII.

### 2.4. Role of IPI in Phage–Bacterial Host Arms Race

The determination that IPI was an inhibitor of T4 genome restriction cleavage originated from a puzzling observation by Lindsay and Kenneth Abremski: in the absence of IPI, the *E. coli* strain CT596 restricted T4 growth [35,36]. The basis for this phenomenon and IPI’s function was finally solved when it was shown to have an anti-restriction function against restriction endonucleases (e.g., CT and UT enzymes) found in certain uropathogenic *E. coli* strains [37,38]. In these strains, there are specific Type IV restriction enzymes able to cleave the hypermodified T4 DNA, which contains glucosylated hydroxymethylcytosine. However, these enzymes are not normally able to cleave the wild-type phage DNA during infection due to their inhibition by IPI. The structural analyses of IPI showed that it folds into a beautiful, small α-helical and β-sheet structure that would facilitate its passage through the phage portal (about 30 Å in diameter) while still folded [39]. Taken together, IPI and the CT enzyme represent a perfect example of the attack and defense mechanisms that co-evolve between bacteriophages and their hosts [39].

### 2.5. Proteolytic Maturation of T4 Head: A Cascade of Protein Conformational Changes

**Figure 3:**
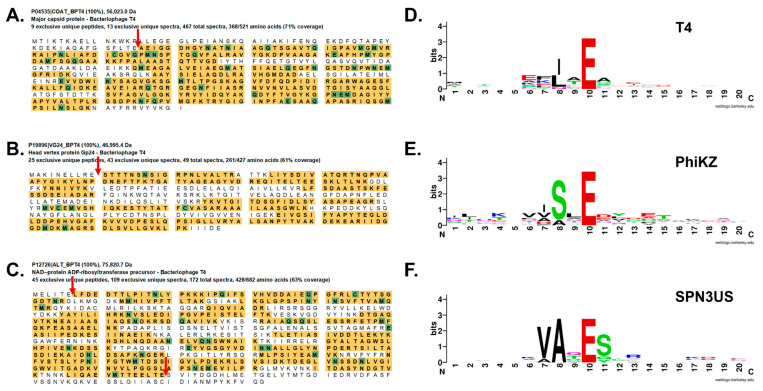
Proteolytic cleavage of head proteins by the prohead proteases of T4 and giant phages. Mass spectral protein sequence coverage of T4 head proteins (**A**) gp23, (**B**) gp24 and (**C**) Alt, with their respective gp21 cleavage sites indicated by red arrows. Giant phages ϕKZ and SPN3US have diverged homologies to T4 gp21 and also cleave their head proteins *C*-terminal to a glutamyl residue after a short sequence motif. Protein sequence logos of the regions flanking the prohead protease cleavage sites in head proteins in (**D**) T4, (**E**) ϕKZ and (**F**) SPN3US. Sequence logos were created using WebLogo (https://weblogo.berkeley.edu/logo.cgi, accessed on 12 February 2022).

Prior to DNA packaging, there is a transformation of the spherical T4 prohead caused by the proteolytic cleavages and conformational rearrangements of both shell and internal proteins, causing a dramatic remodeling of both the external and internal environments of the capsid [40,41] (Figure 1C). These cleavages are performed by gp21, a serine protease [42] with homology to the prohead proteases of other tailed phages (at varying levels of divergence) and Herpesvirus [43,44,45]. The cleavage specificity of gp21 was first hinted at by the determination that gp22 is cleaved at a glutamyl–alanyl bond [46]. Additional studies confirmed that the substrates of gp21 are cleaved *C*-terminal to a glutamyl residue after a surprisingly short sequence motif L/I-X-E [4,42,46] (Figure 3).

The general locale in the polypeptide chain where gp21 cleavage occurs broadly correlates with the functions of its substrates. The scaffold protein gp22, and two other prohead core proteins gp67 and gp6, which also likely have roles in head size determination [47], are cleaved into multiple fragments by gp21. The resulting peptide fragments of the core proteins and the propeptide regions of other proteins are mostly removed from the head, presumably exiting it via pores in the immature capsid shell, and/or the portal channel. The removal of the major core proteins (except gp22*, IPIII and Alt) creates volume within the head later held by the packaged genome. In contrast to the core proteins, proteins that are components of the mature virion have *N*-terminal propeptides ranging from 7 to 65 residues in length removed, for Alt and gp23, respectively. The removal of the gp23 propeptide is notable, since its removal drives the conformational changes of the mature fragment, gp23*, which facilitate outer shell expansion, even in the absence of DNA packaging [48]. This expansion is a fascinating example of protein folding dynamics that increase the capsid volume available for DNA packaging by ~65%, increases shell stability and enables the binding of the Small outer capsid protein (Soc) and Hoc to the outer lattice [49,50,51].

### 2.6. Targeting of Proteins into the T4 Phage Head

The IP proteins have short 10–19-residue long *N*-terminal propeptides that, during assembly, target these proteins into the core to which they have affinity. The proteolytic removal of each propeptide releases the mature fragment, and therefore their interactions with the core proteins. The role of these propeptides was determined via an ingenious approach whereby the internal protein IPIII was fused at the genetic level to foreign proteins [52] (see Section 2.7). The IP propeptides were given the apt name Capsid Targeting Sequences (CTSs) after truncation experiments showed that only the first 10 amino acid residues of IPIII were required for its incorporation into the head [53]. The specificity of the 10 CTS residues of IPIII was tested with amber mutations at the codon positions 2, 4, 7, and 8, respectively. Then, infections were performed in a collection of different amber suppressor strains, which translate the amber codon with Ala, Gly, Ser, Gln, Leu, Tyr and Phe, respectively [53]. This analysis showed that the capsid targeting is mainly located in the N-terminal part of CTS, particularly at residues 4 and 7, whereas the processing of CTS is located at residues 8 and 10. Intriguingly, an aspartyl residue at position 10 inhibits processing, but still allows the targeting into the capsid. Based on these analyses of IPIII, the propeptides of the other IPs are inferred to have CTS roles, especially since the IPII propeptide is identical to that of IPIII, and there is sequence similarity between the IPIII and IPI propeptides. Based on the sequence divergence between the mature regions of T4′s IP proteins, as well as the array of proteins packaged within the capsids of related phages, the CTS may represent an evolutionary mechanism of T4-like phages to incorporate various proteins within their capsids.

For quite some time, gp21 was the only known T4 head protein expected to undergo *C*-terminal processing [42] until recent mass spectral analyses revealed that Alt also undergoes *C*-terminal processing by gp21 [54] (Figure 3). That both of Alt’s termini are propeptides suggests the possibility that both termini of gp21 (and possibly other proteins) are also cleaved, since gp21 also has a sequence close to its *N*-terminus that conforms with its cleavage logo (LIE-9). The autocleavage of gp21 is one of the major gaps in our understanding of the role of gp21, despite extensive research. Possibly, gp21 propeptides act to target the enzyme into the prohead, akin to the CTS of the IP proteins. Their cleavage then removes gp21-core protein interactions, and in doing so facilitate head maturation. It is also conceivable that autocleavage is linked to protease activation, which could be a reason for the very few apparent determinants for cleavage by gp21. An additional puzzle regarding gp21 is the second start codon (Met45) within its gene that causes the expression of a short gp21s, which is also essential for T4 head assembly [55].

### 2.7. Fusion Proteins and Phage Display with Bacteriophage T4

**Figure 4:**
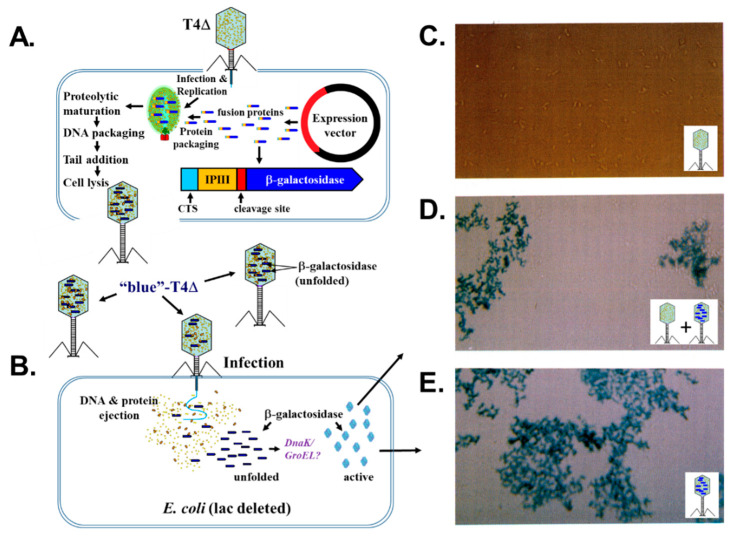
Delivery of β-galactosidase into *E. coli* via the T4 expression-packaging-processing system. (**A**) Scheme of the incorporation of β-galactosidase produced by an expression plasmid into the T4 head via its fusion to the T4 internal protein IPIII and (**B**) subsequent infection of the progeny “blue”-T4Δ phage and ejection of β-galactosidase into an infected cell. The T4Δ genotype is Δe-ΔIPIII-ΔIPII-alt−s12 [32,52]. Light microscopy of *E. coli* cells infected with (**C**) T4Δ, (**D**) T4Δ and “blue”-T4Δ and (**E**) “blue”-T4Δ after incubation with X-Gal. (B–E) adapted from Hong and Black (1993).

The abovementioned ingenious approach to target non-T4 proteins into the capsid was to fuse the internal protein IPIII at the genetic level to foreign proteins such as β-galactosidase (Figure 4), β-globin, *Eco*RI endonuclease, luciferase, green fluorescent protein (GFP) or the nuclease from *Staphylococcus aureus* (SN) [52,53]. The fusions to the *C*-terminus of IPIII, or only its CTS, to the *N*-terminus of these foreign proteins led to their packaging into the phage head and subsequent delivery into the host cell upon infection. Quantitative estimations revealed that 20 to 200 fusion proteins per phage particle can be packaged [56]. This was also nicely demonstrated with a CTS–GFP fusion, leading to phages with heads packaged with GFP [56]. GFP phages were only observed to fluoresce when their infections were performed at 20–25 °C; at 37 °C, the phage particles contained non-fluorescent GFP, consistent with earlier studies showing that GFP fluorescence in *E. coli* was sensitive to temperature. However, when the phages containing non-fluorescent GFP infected host cells at 25 °C, the host cells exhibited fluorescence, suggesting a refolding event for GFP after ejection through the portal and tail.

The fusion of IPIII to SN resulted in up to 200 copies of packaged nuclease per phage. In the phage head, the fusion protein is processed and can be activated, leading to the nucleolytic digestion of the phage DNA if Ca^++^ is added [56]. The fragmentation of the phage DNA leads to the loss of infectivity of the phage within minutes. Amazingly, the SN enzyme inside the capsid can be activated by the Ca^++^ added from the outside of the capsid, suggesting that the ions easily pass through the capsid shell. It also suggests that SN can diffuse around inside the head despite the condensed state of the packaged phage DNA.

Another interesting area pioneered by Lindsay Black deals with the fusion proteins of externally exposed T4 head proteins that can be used for phage display. At first, *C*-terminal fusions were made with the non-essential Soc protein. Soc binds to gp23* between the hexameric capsomers on the outside of the phage head. In contrast to the filamentous phage display system [57], the Soc display has no size limitations and the 312 amino acid long VP1 of poliovirus was successfully displayed [58]. The surface area of one phage head allows the display of around 1000 copies of Soc-fusion proteins without impeding the assembly of the phage. Purified T4 phage with a Soc-V3 fusion protein of the human immunodeficiency virus HIV was used to immunize mice, which successfully developed an IgG response [58]. Lindsay continued to employ the use of exterior fusion proteins to test various questions, not only with Soc, but also Hoc, with the latter being used to test the rotary portal model, as noted above.

Soc and Hoc were used to display a library of randomized peptide fusions to seek out proteins that interact with TerL (gp17). Proteins known to interact, such as gp20 and the single-stranded DNA binding protein gp32, but also new binding partners were identified [59]. Notably, among the latter was gp55, which displays the most sequence matches, and led to co-immunoprecipitation studies using both anti-gp55 and anti-gp17 antibodies. The binding of gp55 to a gp17–intein fusion protein confirmed there were physical interactions between gp17 and gp55, suggesting that gp55 has a role in T4 DNA packaging in vivo [59]. These findings were remarkable, as gp55 acts as a sigma subunit of *E. coli* RNA polymerase, which is highly modified (e.g., ribosylated [60]), and transcribes the T4 late genes from short −10 promoters that have no corresponding −35 sequence. Since gp55 also interacts with the T4 sliding clamp (gp45) and the late transcription co-activator (gp33), this highlighted a potential mechanistic coupling of packaging, transcription and DNA replication (termed DNA transaction) events late in T4 infection [59]. In vitro packaging assays to assess the impacts of these proteins on T4 DNA packaging supported a requirement for gp55 in the handling of the “endless” concatemeric DNA that is present in the cell late in infection, as is seen in vivo with a close mechanistic connection between DNA packaging and DNA replication-dependent late transcription in T4 [8] (Figure 1).

## 3. The Ever-Expanding Value of T4 Phage for Elucidating Head Assembly Mechanisms in Other Phages

### 3.1. Billions of Head Assemblies Each Day Defined by Studies on T4 

It was well known for many years that phages related to T4, such as T2, T6 and the RB- phages isolated by Rosina Berry, are present in the environment [26,61]. However, it was only after phage genome sequencing became routinely used to characterize new phages that the abundance and prevalence of T4-related phages around the globe could be truly appreciated [62,63,64,65,66,67]. This revelation meant the fundamental studies on T4, many themselves paradigm-shifting discoveries in molecular genetics, emerged to be of a much broader impact than ever imagined. The wealth of knowledge on T4 gene function facilitated the annotation of new, related phage genomes with relative ease. For example, a predicted gene product in a new phage that has homology to T4 gp17 can be annotated as the large terminase and inferred to have a general genome packaging function without any wet-bench research. In addition, the sequencing of phages related to T4 revealed that the major T4 head morphogenesis genes (e.g., gp17, gp20, gp21, gp22 and gp23) are highly conserved and an integral component of what is considered the core genome among T4-related phages [68], perhaps not so unexpected considering the essential status of those genes in T4 [26]. 

In contrast to the highly conserved head core genes, the genome analyses of related phages have evidenced that IPI, IPII and IPIII are not highly conserved. However, many, such as T4D, T2L and T6, have other, related basic proteins with CTS-like sequences that may substitute their function [69]. The lack of strong conservation of IP proteins may well reflect variations on the evolutionary arms race between different bacterial host-restriction systems and infecting phages [39]. An additional layer of complexity in this battle between phage–host combinations are the epigenetic modifications of the DNAs of T4-related phages. Phages closely related to T4 all have hmC, which is similarly synthesized by a phage-encoded thymidylate synthase homolog (gp42 in T4 [70,71]) prior to its incorporation into the genome by their DNA polymerase (gp43 [72]). This hmC is further modified by glucosyltransferases that, in different phages, results in their having different amounts of glucosylated hmC residues, and there are variations in both the linkages and the sugar moieties. For instance, 100% of T4 hmC is glucosylated, of which 70% is alpha- and 30% is beta-stereospecific glucosyl hmC [73]. In contrast, about 75% of the hmC in T2 and T6 is glucosylated, of which 70% and 5%, respectively, is transformed to β-1,6-glucosyl-α-glucose (gentiobiosyl) hmC [74]. The recent identification of arabinosyl-hmC (ara-hmC) in RB69 DNA suggests the arsenal of sugar modifications in T4-related phages could be much broader than once realized [75].

### 3.2. Shared Features of Head Maturation between T4 and Phages with Larger Icosahedral Capsids

For many years, the T4 virion was assumed to represent the upper ceiling of tailed phages’ structural complexity being formed from 36 different proteins. The isolation of the giant phage ϕKZ infective for *Pseudomonas aeruginosa*, from the sputum of a patient with a respiratory illness in Russia, heralded that this ceiling could go higher [76,77]. The freeze–thawing of particles prior to TEM revealed an unprecedented feature, a cylindrical proteinaceous structure, the inner body or IB, within a large capsid around which the genome was spooled [78]. Cryo-EM analyses revealed the ϕKZ genome (280 kb) was packaged into a capsid, ~120 Å in diameter, with a T = 27 architecture [79,80]. These studies also highlighted that despite its odd internal inner body, the ϕKZ capsid shell had conserved structural elements observed in other tailed phages. The ϕKZ capsid shell is formed of hexameric blocks of a major capsid protein with the canonical HK97-fold, and houses a genome densely packaged in layers with ~24 Å spacing [80]. Initially, there appeared to be no similarity at the sequence level between ϕKZ and T4, but it was noted that a protease akin to T4′s gp21 was likely responsible for the cleavage of ϕKZ’s major capsid protein gp120 and several other virion proteins identified by *N*-terminal sequencing [79]. Subsequent mass spectral analyses of the ϕKZ head confirmed it comprises more than double the number of different proteins than T4, and the sequence coverage of these proteins indicated that additional ϕKZ virion proteins likely undergo cleavage [81].

The isolation of the phage 201ϕ2-1, infective for *P. chlororaphis*, from a soil sample in Texas was one of the first indicators that large phages related to ϕKZ might be widespread geographically [82]. Cryo-EM and proteomic analyses of 201ϕ2-1 revealed it had a large mass of proteins within its capsid, and two multi-subunit RNA polymerases (RNAPs), one of which is part of the virion (vRNAP) [83]. Studies on other giant phages, including EL and Lin68, have shown that these characteristics are hallmark features of these phages [84]. Proteomic analyses of the 201ϕ2-1 virion identified 89 different proteins, of which 19 were found to undergo processing in a manner analogous to the cleaved T4 head proteins [85]. A sequence logo of the 29 cleavage sites in those 201ϕ2-1 proteins showed all occurred *C*-terminal to a glutamyl residue with extremely few other cleavage determinants.

Due to his long interest in T4 head maturation and its IP proteins, the extensive proteolysis in phage 201ϕ2-1 intrigued Lindsay. Based on the T4 precedents, it was not far-fetched to infer that the cleaved proteins of 201ϕ2-1 must be head proteins, and at least some of them would be responsible for the formation of the protein mass within its capsid and/or ejected into the host cell (ejection or E proteins). That some phage head proteins would be ejected into the host was strongly supported by the presence of the vRNAP, which in ϕKZ was shown to be responsible for transcription early in infection [86]. The questions regarding the head proteins of these large phages led to the cryoEM reconstruction of the ϕKZ IB by Weimin Wu with Niaqian Cheng and Alasdair Steven. Their analyses showed that the IB had a surprisingly ordered structure, and the volume it occupied within the capsid was greater than that of the entire capsid of many smaller phages [87]. Biochemical and mass spectral analyses of the ϕKZ head, including particles in which DNA packaging was prevented by the use of 9-aminoacridine (an inhibitor of T4 DNA packaging [88]), revealed it had a similar number of processed head proteins as 201ϕ2-1 [89]. The ϕKZ head proteins were all cleaved *C*-terminal on a glutamyl residue at the motif S/A-X-E (where X is any amino acid), highly reminiscent of the cleavage motif of T4, and no other identifiable cleavage requirements (Figure 3). In addition, several highly abundant and cleaved ϕKZ proteins were shown to be associated with the DNA, supporting their being excellent candidates for major components of the IB structure.

Until that point, the protease responsible for these cleavages in ϕKZ and its relatives had remained elusive, but an increase in the sequences in the databases facilitated HMM-based searches that produced a diverged match between ϕKZ gp175 and T4 gp21 [89]. The plausibility of the match was supported by the alignment of the predicted catalytic residues. In addition, gp175, like its homolog in 201phi2-1 (gp268), had undergone *C*-terminal processing, potentially a co-incidental similarity to T4 gp21, or potentially evidence of an anciently-derived step in head maturation. Confirmation that gp175 was the driver of ϕKZ head maturation, a process estimated to involve at least 6000 cleavages per capsid, was obtained via assays of recombinant purified gp175 against IB protein substrates [90]. These assays provided evidence that there was a built-in redundancy for maturation, i.e., propeptide regions contained multiple cleavage sites to ensure that they are removed, or mostly removed, from the head. Based on the presence of sequences that conform to the cleavage motif in many homologous proteins, proteolytic cleavage is likely a common phenomenon in related phages during head maturation.

**Figure 5:**
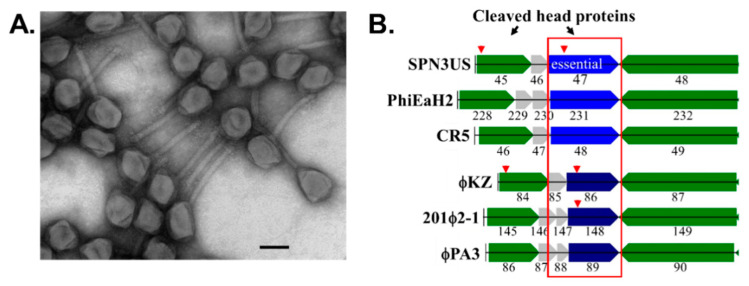
The first mutant of *Salmonella* phage SPN3US, *47*(am1)—isolated by Lindsay Black—has an amber mutation in a low copy number head ejection protein gp47. (**A**) Transmission electron micrograph of *47*(am1) grown under non-permissive conditions shows particles with an apparent wild-type phenotype, but are non-viable (reversion rate < 1 × 10^−5^). Space bar represents 100 nm. (**B**) The gp47 gene is located in cluster of head genes conserved in related giant phages, including *Erwinia* phage PhiEaH2, *Cronobacter* phage CR5 and *Pseudomonas* phages ϕKZ, 201ϕ2-1 and ϕPA3. Red arrowhead indicates a gene product undergoes cleavage by the prohead protease. Images adapted from Ali et al. [54].

**Figure 6:**
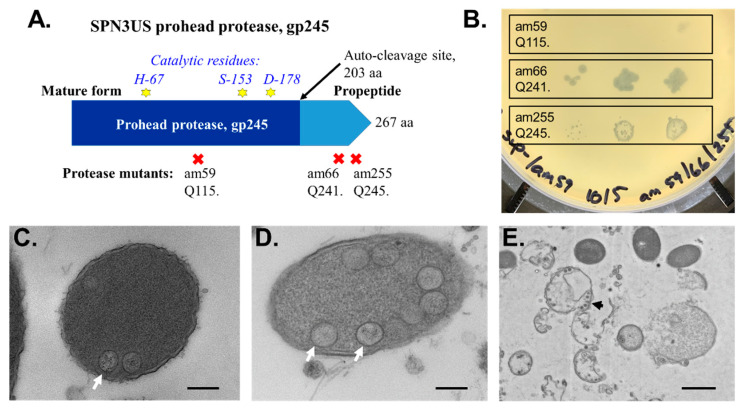
Proteolytic cleavage of head proteins by the prohead protease is essential for head maturation in *Salmonella* phage SPN3US. (**A**) Scheme of the SPN3US prohead protease gp245 showing the locations of catalytic residues. (**B**) Cross-plating of SPN3US protease mutants spotted on a lawn of a non-permissive strain seeded with mutant am59, showing complementation (intragenic recombination) with am66 and am255. (**C**–**E**) Electron microscopy of thin sections of a non-permissive strain of *Salmonella* infected with (**C**) the wild-type phage at 10 min post-infection and (**D**,**E**) am59 at 90 min post-infection. Examples of proheads attached to the host inner membrane are indicated with white arrows. (**E**) In the absence of proteolysis, proheads were observed still attached to the inner membrane after cell lysis (black arrow), which was delayed relative to normal. Space bar represents 200 nm (**C**,**D**) and (**E**) 1 µm.

The isolation of SPN3US, infective for *Salmonella enterica* serovar Typhimurium [91], demonstrated that large phages with hallmark features of ϕKZ, but with hosts outside the genus *Pseudomonas*, existed. SPN3US has two multi-subunit RNAPs and a T = 27 capsid that contains a bolus of E proteins and is transformed by proteolytic maturation [54,92,93]. Our focus switched to SPN3US, since its host is amenable to genetic analyses, and Lindsay led the foray into SPN3US mutant isolation using the same random mutagenesis approach used for the isolation of many T4 amber mutants [94,95] (Figure 5). The sequencing of SPN3US mutants has facilitated the identification of essential genes in SPN3US, including those required for transcription, DNA replication and repair, and also genes required for the formation of its virion [54,95,96]. As in T4, the SPN3US mutants themselves are useful for studying the functions of novel giant phage genes, and have a broader relevance due to the increased isolation of long-genome phages with homologous proteins (see Figure 5 for a few examples). Mutant analyses have also highlighted the ancestrally-derived similarities in head assembly between SPN3US, and T4, e.g., SPN3US heads assemble on the host inner membrane and only after proteolysis are released from it to participate in DNA packaging [92] (Figure 6). Although keenly aware of the differences between SPN3US and T4, it was such overarching similarities that caused Lindsay to note—with great satisfaction—on multiple occasions: “It is just like T4”.

## 4. Conclusions

It was clear to those of us who had the privilege of knowing Lindsay Black that he was an absolutely brilliant scientist. We have highlighted here just a few of Lindsay’s research achievements with the hope that readers may not only be familiar with his many contributions to the field, but also be inspired to themselves consider working towards answering some of the many questions that still remain regarding T4 head assembly and that of other phages. Lindsay was acutely aware of the important questions that still need to be resolved, whether they regard DNA packaging, internal proteins, core assembly or head maturation. Let us honor him in the quest to resolve each and every one!

## Figures and Tables

**Table 1:** Composition of the T4 prohead and head (adapted from Black, Showe and Steven [4] and Rao and Black [30]).

gp	Function	Prohead, Copy Number	Mature Head, Copy Number	Cleaved?	Essential?
**Outer shell**					
20	portal	12	12	-	+
23	major capsid	930	930	+	+
24	head vertex	55	55	+	+
Hoc	decoration protein	0	155	-	-
Soc	decoration protein	0	810	-	-
**Internal**					
21	prohead protease	72	3	+	+
22	scaffold	576	115 (2.5 kDa fragment)	+	+
67	core component	341	139 (3.9 kDa fragment)	+	+
68	core component	240	0	-	+
Alt	ADP-ribosyltransferase	40	40	+	-
IPI	internal protein I, Type IV restriction enzyme inhibitor	360	360	+	semi
IPII	internal protein II	360	360	+	-
IPIII	internal protein III	370	370	+	-

## Data Availability

Not applicable.
